# Dopaminergic neuron loss in mice due to increased levels of wild-type human α-Synuclein only takes place under conditions of accelerated aging

**DOI:** 10.1038/s41598-024-53093-1

**Published:** 2024-01-30

**Authors:** Ana Perez-Villalba, María Salomé Sirerol-Piquer, Raúl Soriano-Cantón, Virginia Folgado, Azucena Pérez-Cañamás, Martina Kirstein, Isabel Fariñas, Francisco Pérez-Sánchez

**Affiliations:** 1 Departamento de Biología Celular, Biología Funcional y Antropología Física, Universitat de València, Valencia, Spain; 2 Instituto de Biotecnología y Biomedicina (BioTecMed), Universitat de València, Valencia, Spain; 3 Centro de Investigación Biomédica en Red de Enfermedades Neurodegenerativas (CIBERNED), Madrid, Spain; 4 Laboratory of Animal Behavior Phenotype (L.A.B.P.), Department of Neuropsychology, Faculty of Psychology, Catholic University of Valencia, Valencia, Spain

**Keywords:** Cell biology, Neuroscience

## Abstract

Understanding the intricate pathogenic mechanisms behind Parkinson's disease (PD) and its multifactorial nature presents a significant challenge in disease modeling. To address this, we explore genetic models that better capture the disease's complexity. Given that aging is the primary risk factor for PD, this study investigates the impact of aging in conjunction with overexpression of wild-type human α-synuclein (α-Syn) in the dopaminergic system. This is achieved by introducing a novel transgenic mouse strain overexpressing α-Syn under the TH-promoter within the senescence-accelerated SAMP8 (P8) genetic background. Behavioral assessments, conducted at both 10 and 16 months of age, unveil motor impairments exclusive to P8 α-SynTg mice, a phenomenon conspicuously absent in α-SynTg mice. These findings suggest a synergistic interplay between heightened α-Syn levels and the aging process, resulting in motor deficits. These motor disturbances correlate with reduced dopamine (DA) levels, increased DA turnover, synaptic terminal loss, and notably, the depletion of dopaminergic neurons in the substantia nigra and noradrenergic neurons in the locus coeruleus. Furthermore, P8 α-SynTg mice exhibit alterations in gut transit time, mirroring early PD symptoms. In summary, P8 α-SynTg mice effectively replicate parkinsonian phenotypes by combining α-Syn transgene expression with accelerated aging. This model offers valuable insights into the understanding of PD and serves as a valuable platform for further research.

## Introduction

Parkinson's disease (PD) is a chronic and progressive sporadic neurodegenerative disease giving rise to a broad spectrum of motor and non-motor symptoms. It is characterized by the gradual degeneration of dopaminergic neurons (DAN) located in the *substantia nigra pars compacta* (SN) that leads to an age-related profound depletion of dopamine (DA) in striatal projections and thereby a dysfunctional nigrostriatal system^1,2^. Affected neurons contain abnormal proteinaceous fibrillar cytoplasmic inclusions called Lewy bodies (LBs) and dystrophic neurites enriched with α-synuclein (α-Syn)^3,4^. While the putative α-Syn biological function in synaptic vesicle DA release and neurotransmission is still debated, its critical involvement in the pathogenesis of PD and other neurodegenerative diseases, collectively known as α-synucleinopathies, has been substantiated by numerous genetic and biochemical studies (for a recent review see Sharma and Burré^5^). Notably, factors such as trans-synaptic connectivity and cell autonomous mechanisms appear to govern the vulnerability of DAN, influencing the progression of synucleinopathy^6^. Additionally, alterations in the dose and conformation of α-Syn are significant factors contributing to disease susceptibility, as evidenced by missense mutations and rare multiplications of the α-Syn (*SNCA*) gene^7,8^. These genetic variants render the protein more prone to misfolding and fibrillization, further increasing the predisposition to PD.

A considerable challenge in the study of PD has been its successful recreation in mouse models. Various approaches have included the generation of transgenic mice expressing normal or mutated human α-Syn, intracerebral injection of LB extracts, in vitro pre-formed toxic α-Syn assemblies, or recombinant adeno-associated virus vector (rAAV)-mediated α-Syn overexpression^9–11^. While some of these methods can induce parkinsonian-like symptoms, most failed to faithfully replicate human PD^10,12^. Specifically, transgenic mice overexpressing wild-type human α-Syn can exhibit certain dysfunctions, but in the absence of overt frank nigrostriatal neurodegeneration^13–16^. On the other hand, models overexpressing mutated forms of α-Syn may produce a phenocopy of the characteristic motor alterations but, if neurodegeneration is present, its pattern occurs mostly in brain areas not specially affected in PD brains and it is largely dissociated from the formation of α-Syn protein inclusion^17–19^.

One plausible explanation for the differential susceptibility of DAN to α-Syn levels and toxicity between rodents and humans is lifespan. Aging is a primary factor in PD pathogenesis, with advancing age, rather than duration of the disease, being the main modifying factor on the phenotypic presentation of the disease^20,21^. When attempting to replicate PD in mice, the relatively short lifespan of these animals compared to humans poses a challenge, as the aging-related components relevant to PD may not have sufficient time to fully manifest, potentially blurring the appearance and expected progression of the disease. Therefore, an attractive strategy is to accelerate the aging process, a phenomenon spontaneously occurring in several strains of senescence-accelerated mice (SAM) that have been selectively bred for the selection of features of premature aging^22^. Mice from the senescence‐accelerated prone 8 (SAMP8) strain exhibit signs of early-onset senility and have a reduced mean life expectancy compared to senescence‐resistant SAMR1 controls^22,23^. Although a specific gene responsible for triggering accelerated senescence in SAMP8 mice has not been pinpointed, it is possible that multigenic single nucleotide variants^24–26^ or alterations in gene expression due to deregulation of epigenetic control^27,28^ play a role in their differential aging dynamics. This mouse model has proven valuable for studying age-related shifts in gene expression patterns and the presence of protein anomalies associated with neurodegenerative diseases like Alzheimer’s disease^29^ and PD^30,31^.

In our effort to enhance the modeling of idiopathic PD as a late-onset disease, we opted to combine two critical factors: α-Syn overexpression and accelerated aging. To achieve this, we conducted successive cross-breeding of a previously characterized mouse line overexpressing wild-type human α-Syn in TH-expressing neurons^16^ onto a senescence-accelerated SAMP8 background. The resulting transgenic mouse line, designated P8 α-SynTg, enabled us to explore the interactive contributions of α-Syn overexpression and accelerated aging to the detrimental changes occurring in the nigrostriatal system and related structures implicated in PD.

## Materials and methods

### Animals

**Figure 1:**
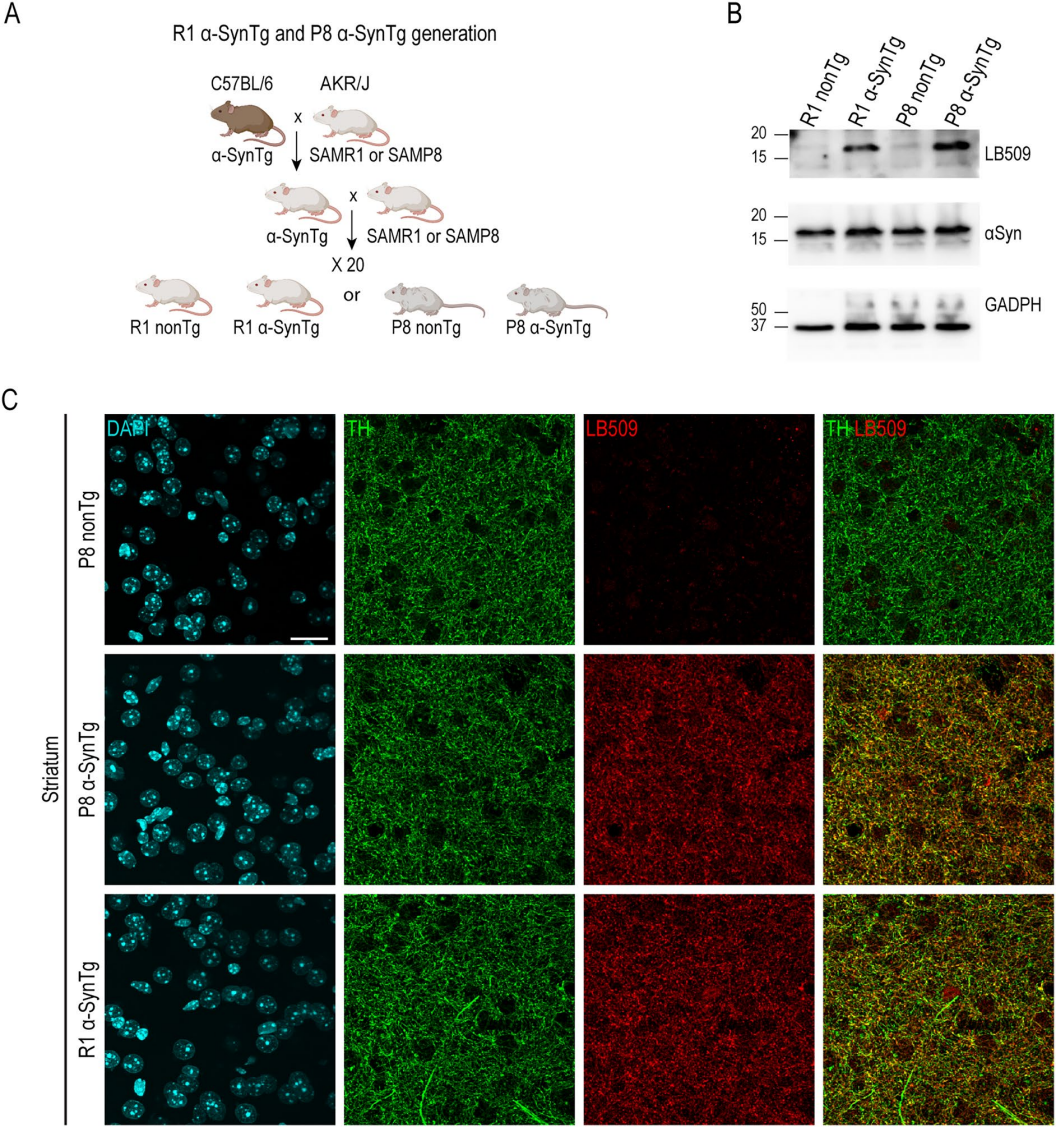
Experimental design and model validation. (**A**) Schematic representation of experimental design for generating distinct mouse groups. (**B**) Representative immunoblots using whole brain lysates from R1 nonTg, R1 α-SynTg, P8 nonTg, and P8 α-SynTg mice, probed with anti-human α-Syn (LB509), α-Syn, and GAPDH antibodies. Western blots were cropped to focus on protein(s) of interest. Uncropped Western blots are shown in Supplementary Fig. S1. (**C**) Representative images of striatum in R1 α-SynTg, P8 nonTg and P8 α-SynTg mice stained with anti-TH and LB509 antibodies. DAPI shows cellular nuclei. Scale bar = 20 μm. (**A**) was created using Biorender.com.

Mice overexpressing α-Syn in the senescence-accelerated SAMP8 (P8) or senescence-resistant SAMR1 (R1) mouse strain genetic backgrounds were generated at the Animal Facility of the Central Service for Experimental Research (SCSIE) of the University of Valencia by crossing a transgenic (Tg) mouse line expressing human wild-type α-Syn under the control of a tyrosine hydroxylase (TH) promoter^16^ for more than 20 generations with P8 or R1 mice (Charles River Laboratories, Barcelona, Spain) (Fig. 1A). Male mice were used for all the experiments. Their body weights at the beginning of the experiments were (average ± SEM): R1 nonTg 31.03 ± 0.75 g, R1 α-SynTg 29.78 ± 0.54 g, P8 nonTg 26.56 ± 0.62 g and P8 α-SynTg 26.90 ± 1.68 g. Experimental groups in SAMP8 strain weighted less than SAMR1 groups at all ages, as it has been previously described^32^.

The presence or absence of the human α-Syn transgene in P8 and R1 animals was confirmed by PCR. Mice were maintained on a 12 h light/dark cycle at a constant ambient temperature of 22 °C with access to water and food ad libitum.

All animal handling and experimental procedures were conducted in accordance with the European Communities Council Directive (2010/63/UE) on the protection of animals used for scientific purposes and were approved by the Research Ethics and Animal Welfare Committee of the University of Valencia (CEEA: A1476445756985). All methods were reported in accordance with the Animal Research: Reporting of In Vivo Experiments (ARRIVE) guidelines 2.0.

### Behavioral procedures

#### Pole test

The pole test, a well-established method used to evaluate movement disorders^11,33^, was conducted on each mouse (n = 6–13). Mice were positioned head-upward on an inclined wooden pole (approximately 10–15° inclination) with a 1 cm diameter and 45 cm length, and a substantial layer of bedding at the base ensured their safety in case of falls. The time taken for the four paws to touch the floor after being placed on the pole’s peak was measured, with a maximum time limit of 90 s. Mice that did not descend the pole head downward in more than one trial were excluded. Additionally, the motor strategy employed during the task was considered, and a Motor Ability Scale (M.A.S.; see Recasens et al.^11^) was used to score mice performance, with 0 points for dragging or falling, 1 point for a time between 7 and 49 s (the longest average duration in the control group while successfully executing the task with the appropriate motor strategy), and 2 points for a time less than 7 s. A higher score indicated better motor performance. The mice underwent three consecutive trials, and the average score was used for statistical analysis. Animals were exposed to the pole test without previous training sessions and with a difference of 6 months between the two evaluation moments to minimize any learning/memory effects.

#### Hind limb clasping test

Hind limb clasping, recognized as an indicator of disease progression in various mouse models of neurodegeneration^34^, was assessed in mice (n = 10–14). The assessment involved lifting the mice by their tails for three trials, each lasting 10 s, while continuous videotaping enabled subsequent evaluation. An experimenter, blinded to experimental conditions, assessed limb position using a 4-stage scale: 0 indicated no change in hind limb position, 1 signified one hind limb retracted close to the body, 2 represented both hind limbs retracted close to the body, and 3 indicated hind limbs retracted and folded tightly along the belly, commonly known as "hind limb clasping." This scale reflects a progressive decline in reflex responses in the hind limbs, a characteristic feature linked to neurodegeneration in motor regions such as the striatum, cerebellum, and spinal cord (reviewed in Lalonde and Strazielle^35^).

#### Balance beam test

The beam motor assay was used to assess horizontal fine motor coordination and balance. Following a previously established protocol^36^, the mice were assessed in traversing a beam measuring 40 cm in length and 1 cm in width. Our evaluation encompassed measuring the time taken to traverse the beam, the number of fallings per experimental group or immobility in each mouse.

### Tissue preparation and immunohistochemistry

Mice were deeply anesthetized (65 mg/kg ketamine and 1 mg/kg medetomidine) and transcardially perfused with 4% paraformaldehyde (PFA) in 0.1 M phosphate buffer (PB). Brains were collected, postfixed overnight in the same fixative, and coronally sectioned at 40 μm with a vibratome (Leica VT1000). For the detection of tyrosine hydroxylase (TH) and human α-Syn, sections were blocked in 10% FBS and 0.1% Triton X-100 and then incubated 24 h with primary antibodies: rabbit anti-TH (1:500, #NB300-109, Novus Biologicals) or mouse anti-human α-Syn LB509 (1:200, #ab27766, Abcam). After several washes with phosphate buffer saline (PBS), sections were incubated for 1 h at room temperature (RT) with appropriate fluorescent secondary antibodies (1:800, #A31570 or #A21206, Invitrogen) and counterstained with DAPI and mounted with Fluorsave (Calbiochem). Images were acquired and processed using an Olympus FluoView FV10i confocal laser microscope and the FV10-ASW 2.1 viewer software. For diaminobenzidine (DAB) staining of catecholaminergic neurons with TH, endogenous peroxidase was inactivated with 3% H_2_O_2_ and 10% MetOH in PBS for 20 min at RT before incubation with primary antibodies. Sections were rinsed in PBS, blocked in 10% FBS and 0.1% Triton X-100 in PBS, and incubated with the anti-TH antibody on an orbital shaker for 24 h at 4 °C. After several washes in PBS, tissue sections were incubated with a goat anti-rabbit IgG biotinylated secondary antibody (1:1,000, #BA-1000, Vector Laboratories) for 1 h at RT. Following washing, sections were processed by a conventional avidin–biotin-peroxidase complex method (ABC, Elite Vector Laboratories). Subsequently, sections were washed and incubated with 0.05% (DAB) (Sigma) in 0.1 M PBS containing 0.01% hydrogen peroxide, mounted on gelatinized slides, dehydrated, and coverslipped for further analysis.

### Unbiased stereological TH-immunoreactive (TH +) neuron counting

Quantitative estimates of the total numbers of TH + neurons in the SN, locus coeruleus (LC), and dorsal motor nucleus of the vagus nerve (DMV) were determined using the unbiased stereological optical fractionator method^37^. Brains were serially coronal vibratome-sectioned at 40 μm, and every sixth to eight section, spanning the entire SN or LC, or every other section spanning the DMV, was processed free-floating for TH-immunoperoxidase as previously described. Sections from groups of mice to be compared were processed and immunolabeled in parallel, with analysis performed blind to the group identities. Nucleus boundaries were determined based on the size and shape of the different TH + neuronal groups and their proximity to nearby fiber bundles and axonal projections^38^. The NewCAST image analysis system (stereology module for VIS; Visiopharm Integration System) was utilized, along with an Olympus BX61 microscope, a DP70 video camera, and a high-resolution integrated motorized stage (Prior ProScan) to outline regions, sample, and count. A 4 × objective was used to delineate brain nuclei, while a 100x/1.4 NA oil immersion objective was used for counting TH + cell bodies. The counting frame size was set at 1,600 µm^2^ (X = 60 µm, Y = 60 µm), with a framing space of 125 µm on X and Y. Guard zones of 5 µm were excluded from both the surfaces and a dissector height of 15 µm was chosen. The sampling fraction was adjusted to ensure a minimum of 150 sampled neurons in each structure per animal. Every cell that appeared within the dissector height was included in the count, as long as they did not intersect with or touch the exclusion lines of the counting frame. The total numbers of TH + cells in the SN, LC, and DMV were estimated using the optical fractionator formula^37^.

### Striatal fiber density measurements

TH + fibers in the striatum were analyzed using the NIH ImageJ 1.49i program from fluorescent micrographs captured with a Nikon Eclipse E800 microscope. Six rostro-caudal coronal images through the dorsal striatum were obtained from each animal using identical microscope settings. Color intensities were converted to a grayscale, with displayed pixel values in the range 0–255, and images were automatically thresholded to isolate the region of interest containing TH + fibers. To account for nonspecific background density, we subtracted the mean pixel value of the corpus callosum from our readings. For each image, we calculated both the relative area occupied by TH + fibers and the integrated density, which was determined by multiplying the mean pixel intensity of the image by the relative area.

### Western blot analysis

For western blotting, we isolated individual hemispheres, excluding the olfactory bulb and cerebellum, from 16-month-old mice. These hemispheres were then lysed in 1 ml of ice-cold RIPA buffer (comprising 50 mM Tris HCl, 150 mM NaCl, 1 mM MgCl2, 1.0% NP-40 (v/v), 0.5% sodium deoxycholate (w/v), 1 mM EDTA, 0.1% SDS (w/v), pH 7.4), and supplemented with phosphatase and protease inhibitors. The protein concentration was determined using a BCA kit (BCA Protein Assay-Kit, Thermofisher). Following this, proteins were separated by SDS-PAGE and subsequently transferred to nitrocellulose membranes (BioRad) using the TransBlot Turbo system. The membranes were blocked for 1 h using 3% skim milk (w/v) in TBS-T. They were then incubated overnight with mouse primary antibodies targeting human α-Syn LB509 (1:500, #ab27766, Abcam), α-Syn (1:500, #610787, BD), GAPDH (1:500, MAB374, Millipore), p-Ser129-α-Syn (1:500, #825701, BioLegend) or β-actin (1:40,000, #A5441, Sigma). After washing, the membranes were exposed to a secondary antibody, goat anti-mouse-HRP (1:10.000, #P0447, Dako) for 1 h and subsequently reacted by chemiluminescence using SuperSignal (Thermo Fisher Scientific). Chemiluminiscence was imaged by an Alliance MINI HD 6 analyzer (UVITEC, UK) and protein expression was calculated by quantifying western blot band intensities using the ImageJ Gel Analyzer plugin.

### Dopamine analysis via HPLC

DA, 3,4-dihydroxyphenylacetic acid (DOPAC), and homovanillic acid (HVA) levels in the striatum were determined using high-performance liquid chromatography (HPLC) coupled to tandem mass spectrometry (MS) as previously described^39^. Immediately after sacrifice, the whole dissected striata of mice were rapidly removed, weighed, frozen in liquid nitrogen (N2) and kept at -80 °C until further use. Upon processing, frozen striatal samples were homogenized in 500 μl of 4% perchloric acid and then centrifuged for 10 min at 10,000×*g* (4 °C). The resultant supernatants were analyzed by HPLC–MS. The chromatographic setup utilized consisted of a Micromass Quatro triple-quadrupole mass spectrometer equipped with a Z-spray electrospray ionization source and an LC-10A (Shimadzu) connected to MassLynx software version 4.1 for the acquisition and analysis of data. We performed the analysis using reversed-phase HPLC with a C18 Mediterranea SEA column (Teknokroma) with 0.5% formic acid and methanol as mobile phase A and B respectively under a gradient program. The flow rate was set at 0.2 ml/min. The analytical conditions for DA, DOPAC and HVA identification and quantification were selected as previously reported^39^.

### Measurement of gut motility (carmine method)

To study total gastrointestinal transit time (GTT), we employed a 6% carmine solution (0.3 ml; Sigma-Aldrich) suspended in 0.5% methylcellulose (Sigma-Aldrich). Each mouse received this solution via oral gavage between 09:00 and 10:00 AM. Subsequently, mice were returned to individual cages, without food deprivation, and monitored for the first observation of carmine red in their stool for a maximum duration of 6 h. GTT represented the time interval between the initiation of gavage and the initial detection of carmine red in the stool. Mice that did not exhibit any dye in their stool, even after a maximum observation period of 360 min, were recorded as having a GTT of 360 min.

### Statistical analysis

Data are presented as either absolute values or normalized (percentual) to the mean levels of control R1 nonTg values and are indicated as the mean ± SEM (standard error of the mean) for n mice. When necessary, data were normalized before statistical analysis to fit Gaussian distribution. Group differences were statistically assessed through a two-way ANOVA, analyzing the main effects of accelerated aging (P8 vs. R1 genetic strain background), α-Syn overexpression (Tg vs. nonTg) and interactions between each factor, followed by a Tukey’s multiple comparison. Significance levels are denoted as *p < 0.05, **p < 0.01, or ***p < 0.001. All statistical analyses were performed using SPSS V. 17 (2008 SPSS Inc., Chicago, IL, USA) or GraphPad Prism 8 (GraphPad Software Inc.).

## Results

### Generation of the SAMP8 senescence-accelerated α-Syn transgenic (P8 α-SynTg) mouse line

To investigate the combined effects of aging and α-Syn overexpression, we created congenic transgenic mouse lines harboring human wild-type α-Syn controlled by a TH promoter^16^ in senescence-accelerated (SAM) strains, P8 and senescence-resistant R1, AKR/J genetic backgrounds. Through over 20 generations of successive back-crossing, we established an age-prone transgenic strain, hereafter referred to as P8 α-SynTg, along with three control lines (R1 α-SynTg, P8 nonTg, and R1 nonTg) (Fig. 1A). Western blot analysis of striatal protein extracts using the LB509 antibody, specific for human α-Syn, confirmed transgene presence in senescent P8 α-SynTg and R1 α-SynTg mice but not in non-transgenic P8 nonTg or R1 nonTg littermates (Fig. 1B). As expected, an antibody recognizing both murine and human α-Syn reacted in all groups, revealing that the human transgene was expressed at low levels, as previously reported for the C57BL/6 background^16^, contributing to a modest increase in overall α-Syn levels (Fig. 1B). Co-immunostaining of brain sections with LB509 and anti-TH antibodies demonstrated transgene expression in the nigrostriatal synaptic terminals of P8 α-SynTg and R1 α-SynTg mice (Fig. 1C), with human α-Syn predominantly localized in TH + nigral axon endings in the striatum, resembling the typical pattern of endogenous α-Syn presynaptic protein.

### Age-related motor coordination deficits in P8 mice are exacerbated by the α-Syn transgene

**Figure 2:**
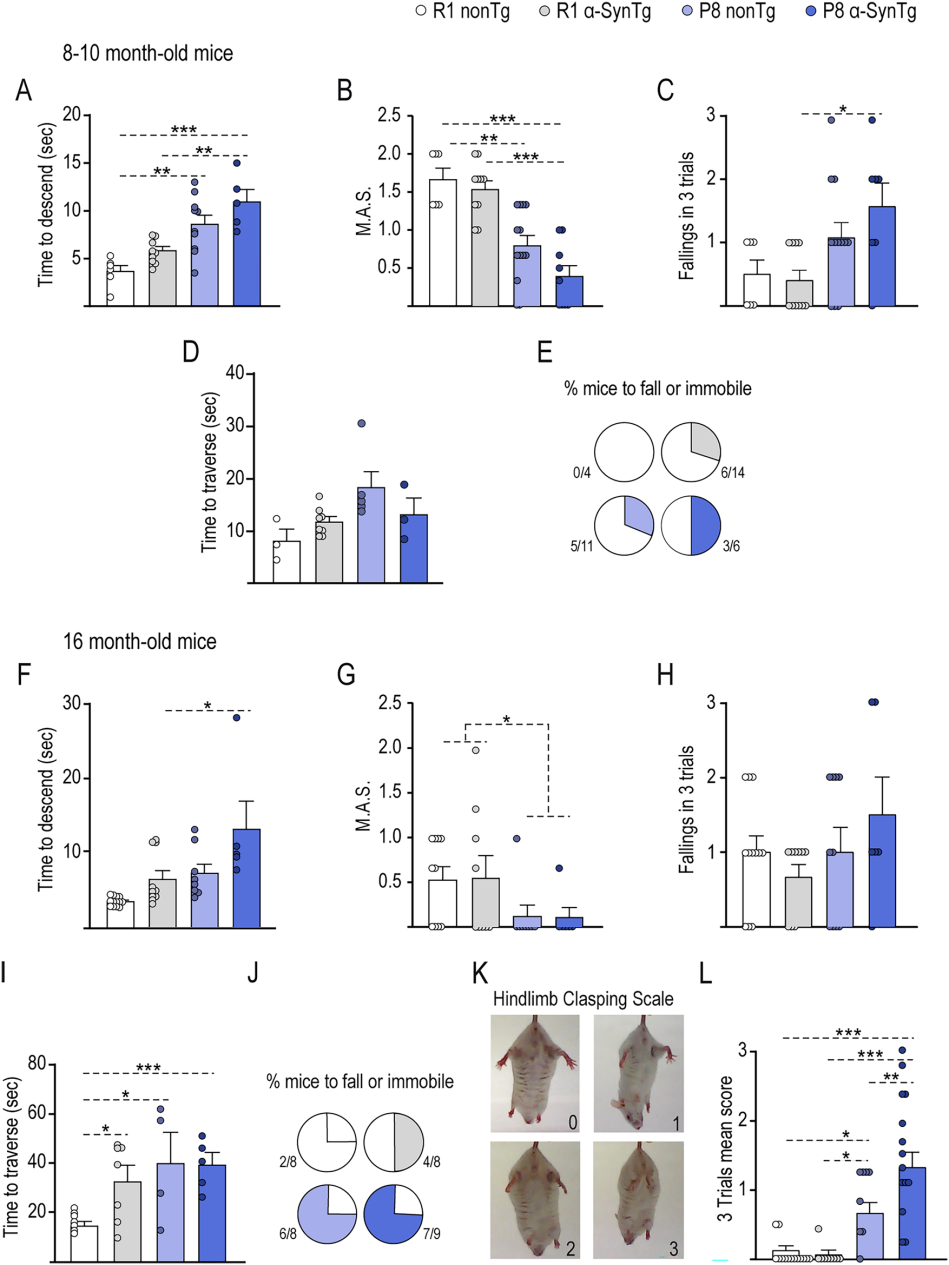
Exacerbation of motor coordination deficits in P8 α-SynTg mice. Graphs display mean ± SEM values for the time spent descending (**A**), the motor ability scale (M.A.S) (**B**), and the number of falls (**C**) in the pole test. Time to travel (**D**) and proportion of fallings or immobilization in the balance beam test (**E**) of 8–10-month-old R1 nonTg, R1 α-SynTg, P8 nonTg, and P8 α-SynTg mice (n = 4–14 mice per group). Graphs show mean ± SEM of time spent descending (**F**), the motor ability scale (M.A.S) (**G**), and the number of falls (**H**) in the pole test. Time to travel the Balance beam test (**I**) and proportion of fallings and immobilization in the test (**J**). (**K**) Representative images illustrating each score in the hindlimb clasping test. (**L**) Graph displaying mean ± SEM values for the hindlimb clasping test scores in 16-month-old R1 nonTg, R1 α-SynTg, P8 nonTg, and P8 α-SynTg mice (n = 5–14 mice per group).

Motor behavior was assessed using the pole test and balance beam test at 10 and 16 months of age, a time point when aging characteristics became more apparent in the P8 α-SynTg group compared to P8 nonTg mice. The pole test evaluates balance and motor coordination of both fore and hind limbs. P8 mice required more time to execute a turnaround and descend the pole in comparison to R1 mice at 10 months of age, and this difference was more pronounced in P8 α-SynTg mice, indicating an interaction between α-Syn expression and accelerated aging (p < 0.001) (Fig. 2A). Furthermore, transgene expression had a more significant impact on coordination in the P8 background compared to the R1 background (p < 0.05). The motor ability scale (M.A.S) also revealed lower task performance rates in the P8 groups (p < 0.001) (Fig. 2B), with the worst performance observed when the transgene was expressed in the P8 background (p < 0.001 compared to R1). The number of falls during the test mirrored these results (Fig. 2C). Then, we evaluated balance and horizontal motor coordination with the balance beam test. Although we did not find a statistically significant difference in the time to traverse the beam (Fig. 2D), P8 α-SynTg mice got the highest rate of fallings and immobilization in the test (Fig. 2E).

At 16 months, the pole test showed that motor coordination deficits were evident in all groups, with P8 mice showing lower ability (accelerated aging, p < 0.05) (Fig. 2F,G) and increased falls (Fig. 2H). P8 α-SynTg mice exhibited the most pronounced deficits (Fig. 2F). These findings indicate that even a modest increase in α-Syn levels exacerbates age-related motor coordination impairments. When we evaluated the balance beam test, all the groups showed a slower performance than the control R1 nonTg group (Fig. 2I) and higher rates of falls and immobilization (Fig. 2J), suggesting that balance and horizontal motor coordination in the beam might be equally affected by the transgene expression or SAMP8 background at 16 months of age.

Due to the inherent variability in motor coordination in advanced age, we conducted an additional assessment using the hindlimb clasping reflex to gauge the severity of motor dysfunction. In this straightforward test, each mouse was gently suspended by its tail for 30 s, and we employed a scoring system to assess the degree of hindlimb clasping: a score of 0 represented both hindlimbs fully extended away from the body with splayed toes, a score of 1 indicated partial retraction of hindlimbs towards the abdomen with splayed toes, a score of 2 denoted both hindlimbs retracted and touching the abdomen, and a score of 3 signified hindlimbs fully clasped over the abdomen (refer to Fig. 2K). The hindlimb clasping reflex has been previously observed in various mouse models of neurodegeneration, including those associated with parkinsonism^33,34^. At 16 months of age, our assessment revealed a substantial deterioration in the hindlimb clasping reflex in the P8 α-SynTg mice (Fig. 2L), which was notably more severe than that observed in the P8 nonTg mice (as indicated by the interaction, p < 0.05). These results underscore the fact that the presence of the α-Syn transgene, especially in the context of accelerated aging, exacerbates motor impairment. Consequently, our findings collectively demonstrate the presence of severe motor deficits in P8 α-SynTg mice, with this deterioration resulting from the interplay between α-Syn transgene expression and accelerated aging.

### Synaptic terminals loss and altered dopamine levels and turnover in the striatum of P8 α-SynTg mice

**Figure 3:**
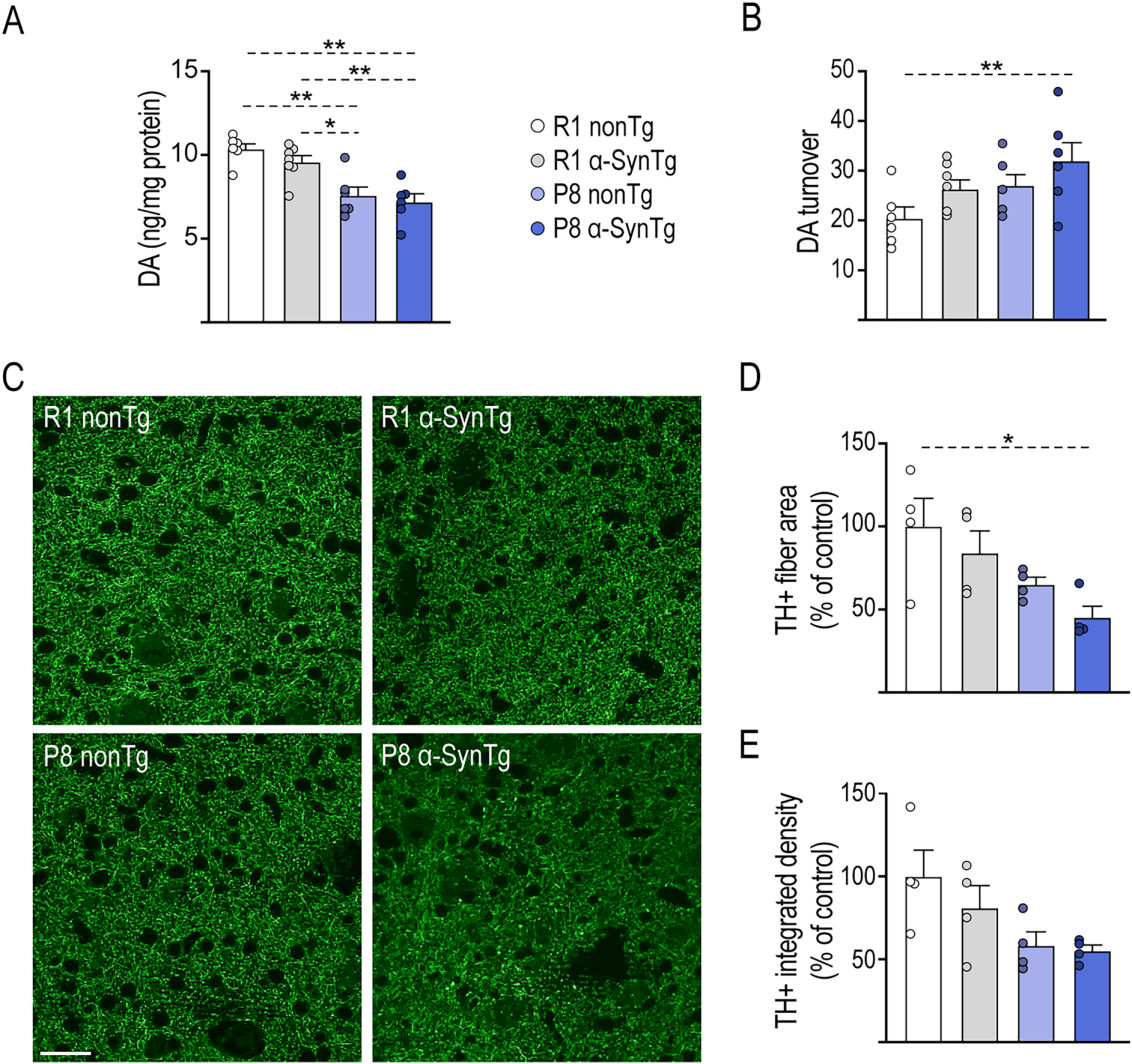
Alterations in DA metabolism and loss of synaptic terminals in the striatum of P8 α-SynTg mice. Graphs show mean ± SEM values for DA levels (**A**) and DA turnover (DOPAC + HVA/DA) (**B**) in the striatum of 16-month-old R1 nonTg, R1 α-SynTg, P8 nonTg and P8 α-SynTg mice (n = 5–7 mice per group). (**C**) Representative images of the striatum in 16-month-old R1 nonTg, R1 α-SynTg, P8 nonTg and P8 α-SynTg mice stained with anti-TH antibody. Scale bar = 15 μm. Graphs depict mean ± SEM of TH + fiber area (**D**) and integrated density (**E**) in the striatum of 16-month-old R1 nonTg, R1 α-SynTg, P8 nonTg and P8 α-SynTg mice (n = 4 mice per group).

To explore whether the observed motor impairments could be linked to a depletion of nigrostriatal dopamine (DA), we conducted an assessment of DA levels and its major metabolites, DOPAC and HVA, in the whole striatum of the four groups of mice using HPLC. At 16 months of age, we noted a substantial decrease of approximately 30% in DA levels in P8 mice compared to R1 mice in the absence of the transgene (p < 0.01) (Fig. 3A). The levels of DOPAC and HVA did not exhibit significant differences among the groups. However, the (DOPAC + HVA)/DA ratio, which provides an estimate of DA turnover, was 30% higher in R1 α-SynTg and P8 nonTg mice and 56% higher in P8 α-SynTg mice compared to the R1 nonTg control group. These differences reached statistical significance, suggesting that both α-Syn expression and accelerated aging contribute to an upregulation of DA turnover in nigrostriatal terminals (p < 0.01) (Fig. 3B). These findings collectively imply that the rate of DA metabolism was accelerated in P8 mice carrying the transgene. To further validate these results, we performed immunofluorescent labeling of TH in sections from the dorsal striatum across all genetic conditions (Fig. 3C) and employed image analysis to quantify the density of TH + fibers. We found a reduction in the area occupied by TH + fibers, with decreases of 16% in R1 α-SynTg, 35% in P8 nonTg, and 55% in P8 α-SynTg mice compared to R1 nonTg controls (Fig. 3D). This reduction was statistically significant due to the influence of accelerated aging (p < 0.05). Similar trends were noted for the optical density of striatal TH + fibers and terminals, providing an indirect estimate of DA levels influenced by the rate-limiting synthesis activity of TH (Fig. 3E). Collectively, these results suggest that accelerated aging plays a predominant role in down-regulating DA levels under the SAMP8 background. Furthermore, the combined effects of accelerated aging and transgene expression contribute to the upregulation of DA turnover, indicating a potential compensatory mechanism at play.

### Moderate α-Syn overexpression leads to neuron loss under conditions of accelerated aging

**Figure 4:**
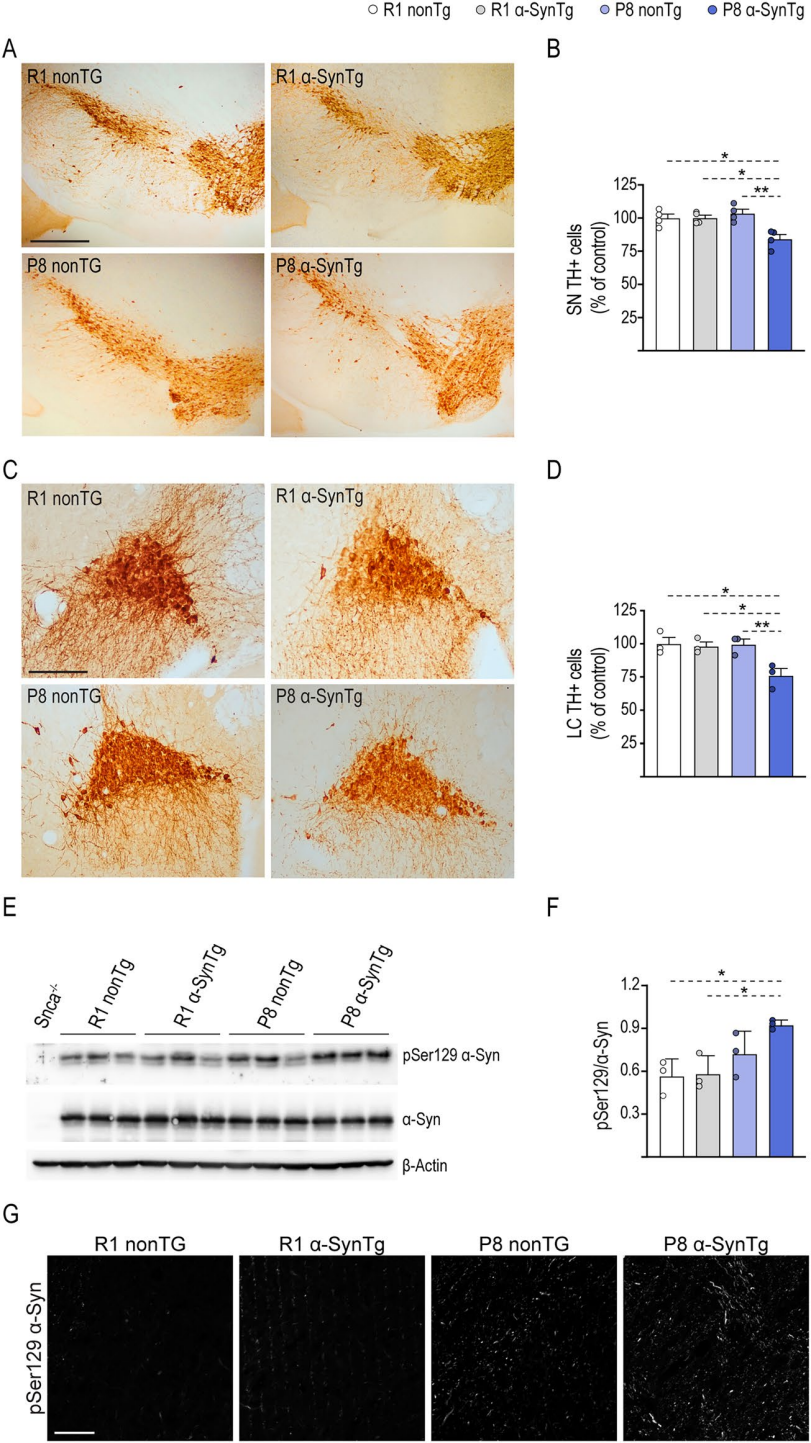
Neuronal loss in the SN and LC of P8 α-SynTg mice. (**A**) Representative images of the SN in 16-month-old R1 nonTg, R1 α-SynTg, P8 nonTg and P8 α-SynTg mice stained with anti-TH antibody. Scale bar = 500 μm. (**B**) Graph displaying mean ± SEM values for the number of TH + neurons in SN of 16-month-old R1 nonTg, R1 α-SynTg, P8 nonTg and P8 α-SynTg mice (n = 4) mice per group. (**C**) Representative images of the LC in 16-month-old R1 nonTg, R1 α-SynTg, P8 nonTg and P8 α-SynTg mice stained with anti-TH antibody. Scale bar = 200 μm. (**D**) Graph shows mean ± SEM values for the number of TH + neurons in the LC of 16-month-old R1 nonTg, R1 α-SynTg, P8 nonTg and P8 α-SynTg mice (n = 3 mice per group). (**E**) Representative immunoblots with anti-p-Ser129-α-Syn, total α-Syn and β-actin antibodies using whole brain lysates from R1 nonTg, R1 α-SynTg, P8 nonTg and P8 α-SynTg mice. Western blots were cropped to focus on protein(s) of interest. Uncropped Western blots are shown in Supplementary Fig. S1. (**F**) Graph shows mean ± SEM of the p-Ser129-α-Syn immunoblot signal normalized to total synuclein (n = 3 mice per group). (**G**) Representative images of the SN in 16-month-old R1 nonTg, R1 α-SynTg, P8 nonTg and P8 α-SynTg mice stained with anti-p-Ser129-α-Syn antibody. Scale bar = 45 μm. *SN* substantia nigra, *LC* locus coeruleus.

To investigate whether the changes in striatal DA content and turnover were associated with an altered complement of DAN in the SN, we first quantified the number of TH + surviving neurons in coronal brain sections from 16-month-old mice by stereological counts (Fig. 4A). Cell count for P8 α-SynTg was reduced in 15% with respect to the other groups of mice, primarily due to the impact of α-Syn expression (p < 0.01) and its interaction with accelerated aging (p < 0.01) (Fig. 4B). Considering that DAN in the SN and noradrenergic neurons in the LC are notably reduced in PD and may share common pathogenic susceptibilities^40,41^, we proceeded to perform TH + cell counts in the LC. Similar to the SN, we observed a moderate but significant loss of neurons, amounting to 24% in P8 α-SynTg mice (Fig. 4C,D). This loss was influenced by accelerated aging, transgene expression, and the interaction of both factors (p < 0.05), further supporting the hypothesis that neuronal deterioration is exacerbated when elevated α-Syn levels coincide with aging. Since phosphorylation of α-Syn at residue Ser129 (p-Ser129-α-Syn) is considered a marker of cellular pathology^42^, our next objective was to assess p-Ser129-α-Syn levels in brain extracts using WB. We observed differences in p-Ser129-α-Syn levels among the groups, with P8 α-SynTg mice exhibiting the highest values (Fig. 4E,F). These variations were attributed to a combination of α-Syn overexpression (p < 0.001) and the genetic background of SAM (p < 0.05). Likewise, immunohistochemistry for p-Ser129-α-Syn exhibited increased reactivity within the SN of P8 groups, particularly noticeable in P8 α-SynTg samples (Fig. 4G).

In conclusion, our findings suggest that modest overexpression of α-Syn in regions associated with PD pathology compromises the integrity of catecholaminergic neurons (both dopaminergic and noradrenergic) specifically when accompanied by accelerated aging.

### Altered gut transit time as an early indicator of PD-like symptoms

**Figure 5:**
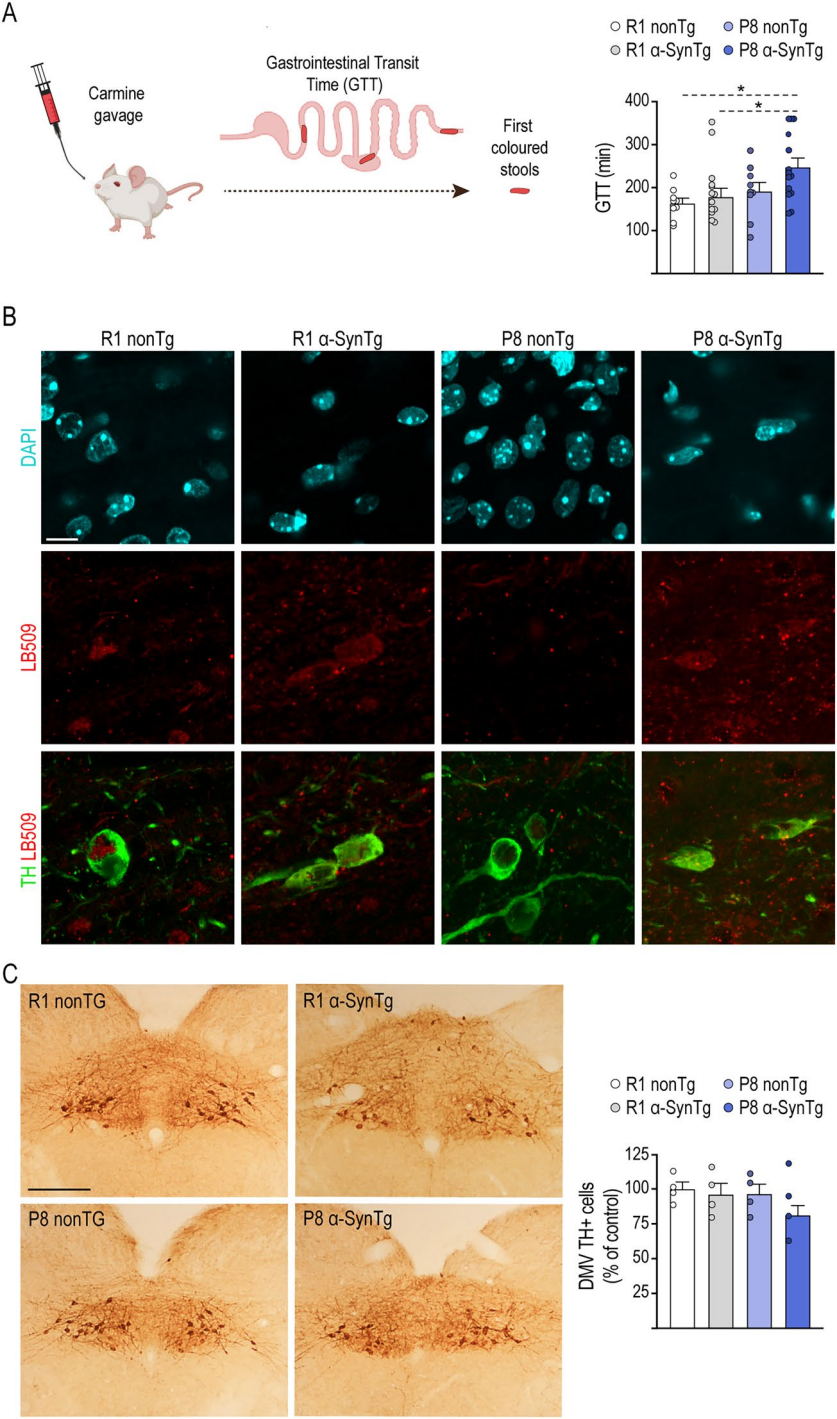
Alterations in gastrointestinal transit time and the DMV in P8 α-SynTg mice. (**A**) Diagram illustrating the experimental design for analyzing GTT in mice. Graph shows mean ± SEM values of GTT in 16-month-old R1 nonTg, R1 α-SynTg, P8 nonTg and P8 α-SynTg mice (n = 9–14 mice per group). (**B**) Representative images of DMV in 16-month-old R1 α-SynTg, P8 nonTg and P8 α-SynTg mice stained with anti-TH and LB509 antibodies. DAPI shows cellular nuclei. Scale bar = 10 μm. (**C**) Representative images of DMV in 16-month-old R1 nonTg, R1 α-SynTg, P8 nonTg and P8 α-SynTg mice stained with anti-TH antibody. Scale bar = 400 μm. Graph displays mean ± SEM values for the number of TH + neurons in DMV of 16 month-old mice (n = 4 mice per group). GTT: Gastrointestinal Transit Time. DMV: Dorsal Motor nucleus of the Vagus nerve. (**A**) was created using Biorender.com.

The involvement of α-Syn pathology in the enteric nervous system and the DMV in the early stages of PD laid the foundation for the Braak staging model of the disease^43^. This concept has been effectively substantiated in animal models^44,45^. In our present study, we employed a sensitive measure of gut function, known as gastrointestinal transit time (GTT), determined by measuring the time taken for mice to pass their first red stool after receiving carmine red through gavage^46–48^. An approximately 50% increase in GTT was observed in P8 α-SynTg mice compared to the other groups, driven by a statistically significant effect of accelerated aging (p < 0.05) (Fig. 5A). It is worth noting that in some P8 α-SynTg mice, the maximum time limit of 360 min was reached without the expulsion of a red stool, a phenomenon observed in three out of fourteen individuals. This suggests the possibility of a more pronounced effect, likely attributable to the combination of accelerated aging and α-Syn overexpression.

The DMV contains the cell bodies of vagal preganglionic neurons that regulate enteric motility^49^. Given that TH + neurons in the DMV may indirectly influence the withdrawal of cholinergic tone to the gut, which can lead to age-related gastrointestinal muscle atrophy^50,51^, we hypothesized that neuron loss in the DMV could be linked to the observed changes in GTT. After confirming the expression of human α-Syn in the DMV of R1 α-SynTg and P8 α-SynTg mice (Fig. 5B), we found substantial reduced numbers of TH + neurons in the P8 α-SynTg group, with a 19% lower count compared to the R1 nonTg group (Fig. 5C).

## Discussion

Parkinson’s disease (PD) remains an enigmatic disorder with a complex etiology, believed to result from a combination of genetic inheritance, aging, and environmental factors, which likely interact synergistically. Overexpression of human wild-type α-Syn is implicated in both rare familial PD cases resulting from SNCA duplications and triplications^8,52^ and sporadic PD^7,53^, whereas loss of DAN has not been observed in transgenic mice that simply overexpress α-Syn. In this study, we introduced the P8 α-SynTg mouse model, generated through several generations of back-crossing transgenic mice overexpressing α-Syn onto a P8 background. P8 mice embody the intricate multifactorial aspects of aging and serve as an excellent mammalian model for accelerated senescence and age-related pathologies^22,54^. The transgenic strain features increased levels of α-Syn in DAN, providing a physiologically relevant means to emulate PD. We selected the TH promoter to drive α-Syn expression due to the pronounced loss of several groups of catecholaminergic neurons in PD, primarily DAN, and the noradrenergic neurons of the LC^55^. Our goal was to replicate the characteristic cell death of these neuronal populations in mice while analyzing the impact of two factors: α-Syn overexpression and accelerated aging, as well as their interaction, to establish this novel mouse strain as a suitable PD model.

In our model, successful specific α-Syn overexpression in TH + neurons and nerve endings was achieved, consistent with previous reports in the parental α-SynTg strain^16^. Unlike other studies using the TH promoter, where overexpression of wild-type human α-Syn did not significantly affect the number of nigral neurons^15,16,56^, the P8 α-SynTg strain exhibited evident neurodegenerative changes leading to motor deficits from the age of 10 months. This result addresses a key limitation in modeling PD through transgenic mice overexpressing α-Syn. In our model, apart from the degeneration of DAN in the SN, LC cell loss exceeded nigral neurodegeneration, resembling the early involvement of the LC observed in PD patients^41^, linked to non-motor prodromal symptoms^57^. Importantly, we identified a significant effect of the P8 background on LC neuron loss of P8 α-SynTg mice, consistent with a prior report of LC neuron loss in the P8 strain^58^. The failure of previous studies to induce catecholaminergic neuron loss in transgenic mice may be attributed to species-specific characteristics, such as the shorter lifespan of mice, potentially delaying DAN degeneration. The P8 background appears to be instrumental in recapitulating human-like conditions that trigger DAN loss. Our findings demonstrate a statistical interaction between α-Syn and senescent status in the establishment of this phenotype.

As in PD, DAN loss in our model was accompanied, and possibly preceded, by degenerative changes in striatal axons and terminals, supporting the concept that α-Syn-induced pathology initially affects axons and terminals before involving cell bodies^59^. Consistent with previous research indicating reduced striatal DA levels in P8 compared to R1 mice^60^, our study revealed a significant contribution of the P8 background to reduced striatal DA markers in P8 α-SynTg mice. Altered DA metabolism is known to be a source of reactive oxygen species and is crucial for neuronal redox homeostasis and viability^61^. Our HPLC analyses, including the assessment of DA metabolites, demonstrated a substantial increase in DA turnover due to the combined effects of the P8 background and α-Syn overexpression. This increased DA turnover may reflect functional changes more accurately than mere loss of total striatal DA, indicating heightened stress on the DA system. Furthermore, partial damage to dopaminergic nigrostriatal projections may lead to compensatory increases in DA synthesis and release from spared neurons, as observed in early PD^57^ and PD animal models^62,63^, being linked to behavioral phenotypes. However, though increased DA turnover may eventually reduce the immediate symptoms of dopaminergic system damage, it may also contribute to its progression through increased oxidative stress^64^. In our model, the increased DA turnover in P8 α-SynTg mice and the inherent high oxidative stress in the P8 background^23,65^, likely exacerbates the oxidative burden on the nigrostriatal system, and further compromises DAN viability. Our results contrast with a previous study that injected recombinant adeno-associated viral vectors (rAAV) to overexpress mutated A53T α-Syn in the SN of mice^66^ and failed to find an additive role of aging and α-Syn overexpression. In that work, C57BL/6 mice exhibited nigral cell loss, while SAMP8 and SAMR1 mice did not. The reasons for these discrepancies are unclear but may relate to the limitations of the rAAV-α-Syn model in mice, which is less robust and more variable than in rats and non-human primates^67^.

The mechanisms underlying the increased dependence of DAN on aging in the context of augmented α-Syn expression are complex, and we are far from understanding them. α-Syn plays a crucial role in maintaining DA homeostasis^68,69^. Its overexpression could lead to cellular damage by disrupting the sequestration of DA in secretory vesicles, potentially triggering oxyradical damage^70^ likely resulting in aberrant stabilized forms of α-Syn^71,72^. Although we did not detect α-Syn aggregation in the SN and striatum of P8 α-SynTg mice, it is plausible that oligomers or other disassembled structures, which are challenging to visualize through conventional immunohistochemistry, could potentially form initially at synapses. This might subsequently evolve, initiating neuronal damage. This progression has been demonstrated using transgenic mouse models designed specifically for directly evaluating the quantity and subcellular distribution of α-Syn oligomers in vivo^73^. In these models, the accumulation of specific oligomers at synapses correlates with a progressive onset of motor decline and the subsequent loss of DAN. An alternative possibility is that the native or misfolded forms of the monomeric protein might also play a role in α-Syn toxicity and the development of PD-related pathogenesis via mechanisms not reliant on aggregation. These mechanisms might include abnormal interaction with membranes, proteins, and other molecules, potential retention within specific cellular compartments, and the disruption of crucial cellular processes.

The increases in oxidative stress^74^ and alterations in α-Syn metabolism with aging likely contribute to an augmented accumulation of oxidized α-Syn variants, which subsequently stabilize during this process. This stabilization could potentially decelerate α-Syn turnover, impacting its flexibility to transition across various distinct conformations. These range from the native states like soluble monomers and membrane-bound tetramers, to soluble transient or structured oligomers, as well as insoluble fibrils and aggregates^75–77^. Furthermore, the presence of oxidized forms of α-Syn could promote additional pathogenic alterations, such as α-Syn truncations^78^, or a range of diverse post-translational modifications^79^. These post-translational changes have been recognized as key determinants of α-Syn functions and aggregation propensity^80,81^. Phosphorylation at Ser129, rarely observed in a physiological state, is significantly elevated in the brains of α-synucleinopathy patients and transgenic PD animal models^43,82^. This modification is thought to play a central role in regulating α-Syn aggregation and neuronal degeneration. Recent research has suggested that Ser129 phosphorylation may inhibit α-Syn aggregation and cytotoxicity, providing protection against higher cell loss^83^. In our model, elevated p-Ser129-α-Syn in P8 α-SynTg mouse brains may have toxic or protective effects on the nigrostriatal phenotype. Determining whether the p-Ser129-α-Syn exists in a monomeric or oligomeric form falls beyond the current study’s scope. Future studies would greatly benefit from the development of tools that allow direct monitoring of protein oligomerization in living cells.

In our model, disruptions in DA metabolism and altered release from spared nigral neurons underlie the motor phenotype, characterized by limb motor coordination and balance deficits, a classical behavioral hallmark in PD models that recapitulates patient symptoms. Additionally, as PD is increasingly recognized as a multi-system disorder, animal models must evolve to encompass the non-motor features of the disease, including those arising from α-Syn pathology in the peripheral nervous system, such as the enteric nervous system^84,85^. Gastrointestinal symptoms, including gastroparesis and constipation, are common non-motor manifestations of PD resulting from gastrointestinal motility disorders^85,86^. In this study, we assessed gut function behaviorally in P8 α-SynTg mice, which exhibited longer GTT compared to other groups, resembling GI dysmotility observed in PD patients. Similar phenotypes have been reported in mice overexpressing human wild-type^45^ or mutated α-Syn^87^. Furthermore, overexpression of A53T α-Syn in the DMV was associated with age-related GI motility impairment^51^, consistent with our findings that a senescent background plays a crucial role in the emergence of gut dysfunction in P8 α-SynTg mice.

In conclusion, the P8 α-SynTg mouse model, characterized by mild human α-Syn overexpression in catecholaminergic neurons and accelerated aging, recapitulates several aspects of PD, including neuronal loss in the SN and LC, motor deficits, and gut dysfunction. This model holds promise for advancing our understanding of PD pathogenesis and developing novel therapeutic strategies.

### Supplementary Information

Supplementary Figures.

## Data Availability

All data generated or analyzed in this study are available from the corresponding author on reasonable request.
