# Supplementary material for: Dopaminergic neuron loss in mice due to increased levels of wild-type human α-Synuclein only takes place under conditions of accelerated aging
Source: Sci Rep. 2024 Jan 30;14:2490. doi: 10.1038/s41598-024-53093-1 (PMC10828501; doi:10.1038/s41598-024-53093-1)
Supplement: Supplementary file 1 — Supplementary Figures. [file 41598_2024_53093_MOESM1_ESM.pdf]

Supplementary information

Dopaminergic neuron loss in mice due to increased levels of wild-type human α-Synuclein only takes place under conditions of accelerated aging

Ana Perez-Villalba[&], María Salomé Sirerol-Piquer[&], Raúl Soriano-Cantón, Virginia Folgado, Azucena Pérez-Cañamás, Martina Kirstein, Isabel Fariñas[*] and Francisco Pérez-Sánchez[*]

Supp. Figure 1

A) Figure 1

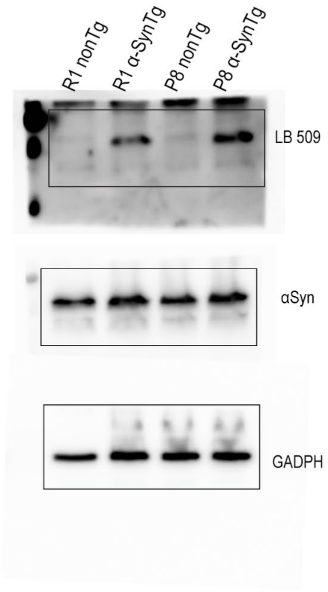

B) Figure 4

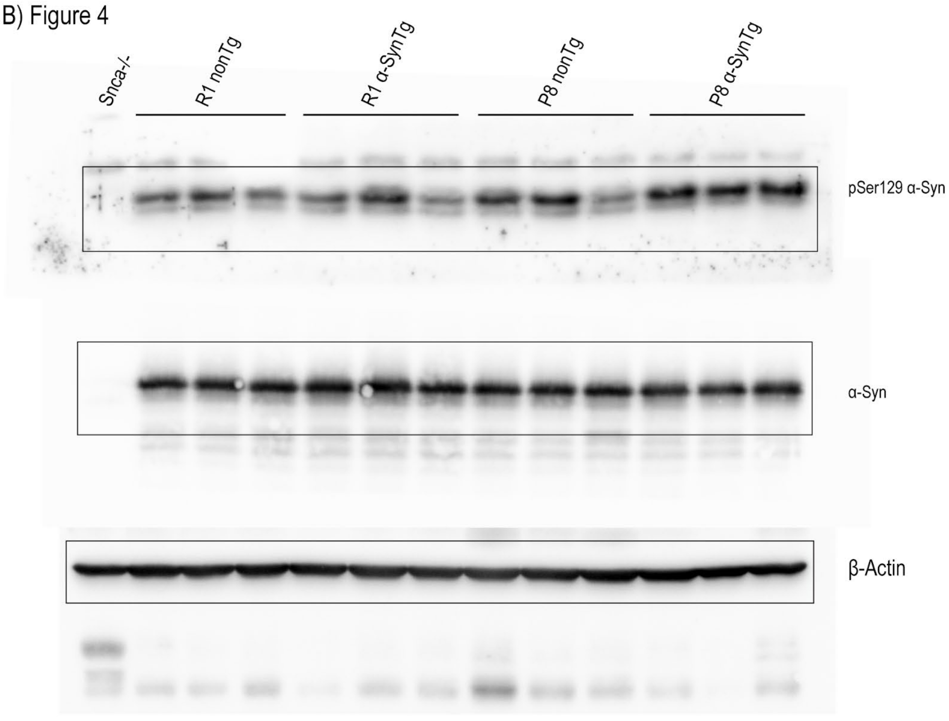

Supp. Figure 1. WB's unprocessed pictures. Figure containing the original chemiluminiscence images presented in figure 1 and figure 4. The squared area corresponds to the cropped area presented in the figures. **(A)** Images from **figure 1**. Representative immunoblots using whole brain lysates from R1 nonTg, R1 α-SynTg, P8 nonTg, and P8 α-SynTg mice, probed with anti-human α-Syn (LB509), α-Syn, and GAPDH antibodies. **(B)** Images from **figure 4**. Representative immunoblots with anti-p-Ser129-α-Syn and α-tubulin antibodies using whole brain lysates from R1 nonTg, R1 α-SynTg, P8 nonTg and P8 α-SynTg mice. **(F)** Graph shows mean ± SEM of the immunoblot signal from (A) (n=3 mice per group). SN: Substantia Nigra, LC: Locus Coeruleus.

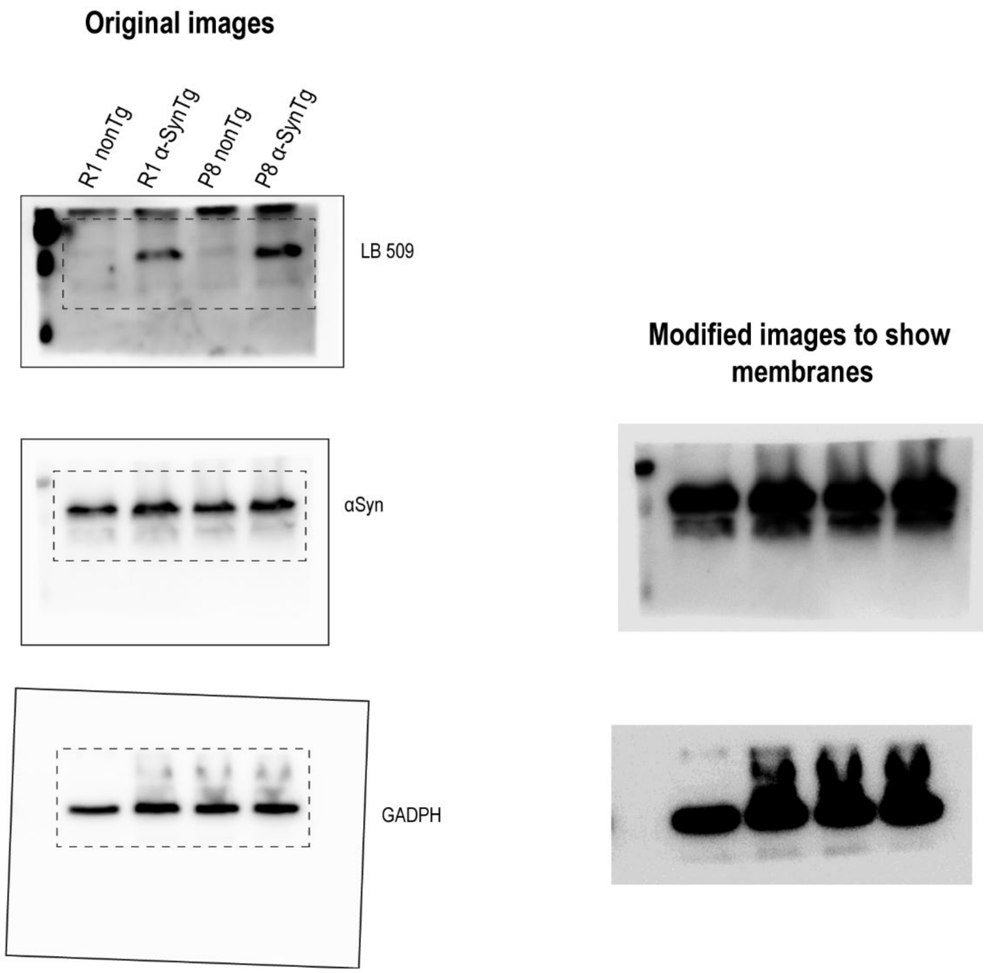

Supp. Figure 2. WB's Figure 1 detailed images. The right panel shows the original pictures obtained with Alliance HD 6 analyzer (UVITEC) depicted with a square. The dotted squares contain the cropped areas presented in the **figure 1**. The left panel represents modified images with Photoshop to show membranes. The staining was performed for LB 509, total αSyn (αSyn) and GADPH.

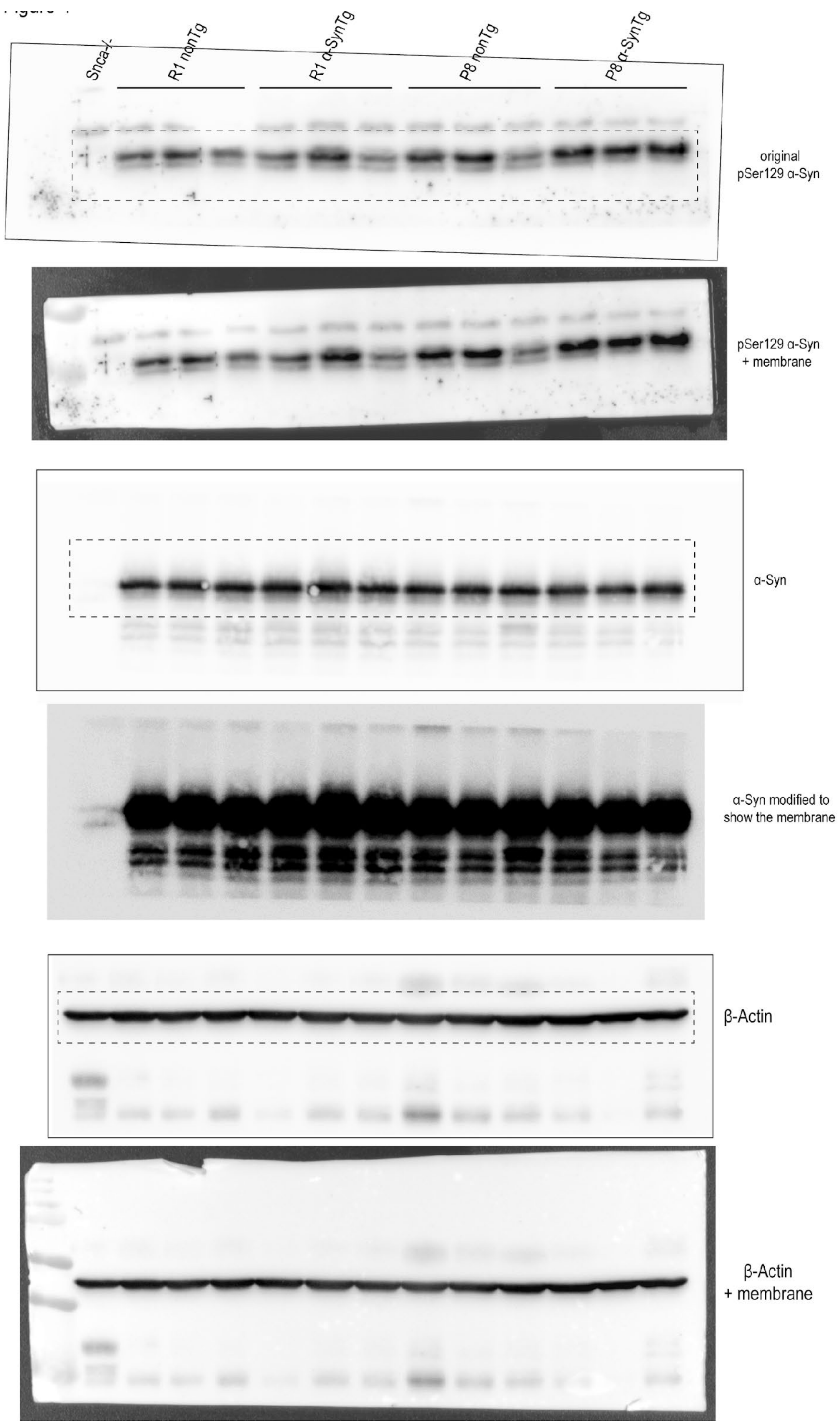

Supp. Figure 3. WB's Figure 4 detailed images. The upper image shows the original pictures obtained with Alliance HD 6 analyzer (UVITEC) depicted with a square. The dotted square contains the cropped areas presented in the **figure 4**. The lower image corresponds to an image fusion of the chemiluminiscence with visible ones or represents modified image with Photoshop to show the membrane. The staining was performed for pSer129 αSyn, total αSyn (αSyn) and β-Actin.
